# Potential mechanisms involving the immobilization of Cd, As and Cr during swine manure composting

**DOI:** 10.1038/s41598-020-73894-4

**Published:** 2020-10-06

**Authors:** Hao-Nan Guo, Li-Xia Wang, Hong-Tao Liu

**Affiliations:** 1 Institute of Geographic Sciences and Natural Resources Research, Chinese Academy of Sciences, Beijing, 100101 China; 2 Northeast Institute of Geography and Agroecology, Chinese Academy of Sciences, Changchun, 130102 China; 3 Engineering Laboratory for Yellow River Delta Modern Agriculture, Chinese Academy of Sciences, Beijing, 100101 China; 4 College of Resource and Environment, University of Chinese Academy of Sciences, Beijing, 100049 China

**Keywords:** Environmental sciences, Biotechnology, Environmental biotechnology

## Abstract

This study aims to investigate the relationship between key physicochemical parameters related to composting process and bioavailability of Cd, As and Cr during swine manure composting through regulating different initial carbon to nitrogen (C/N) ratios (15:1, 20:1, 25:1) and bulking agent types (straw, green waste). Results showed that higher initial C/N ratio of 20:1 or 25:1 and straw as bulking agent were optimal to reduce the bioavailability of Cd, As and Cr (62.4%, 20.6% and 32.2% reduction, respectively). Redundancy analysis implied that the bioavailability of Cd was significantly associated with total phosphorus and total nitrogen, deducing the formation of phosphate precipitation and biosorption might participated in the reaction process, while that of As and Cr were mainly influenced by organic matter (OM), cation exchange capacity (CEC) and OM, CEC, electric conductivity, respectively. A total of 48.5%, 64.6% and 62.2% of Cd, As and Cr redistribution information could be explained by the above parameters. Further correlation analysis revealed that bioavailable As and Cr were negatively correlated with humic acid to fulvic acid ratio. In summary, this study confirms that the mechanisms of phosphate precipitation, biosorption and humification played critical role in reducing Cd, As and Cr bioavailability during swine manure composting.

## Introduction

Livestock manure is regarded as an ideal source of organic fertilizer because it contains a large amount of phyto-nutrients^1^. However, the problem of excessive heavy metals in livestock manure caused by using feed additives, such as Cu, Zn, Cd, As and Cr, can pose serious environmental risks through land application, thus limiting the utilization of livestock manure^2^. Therefore, it is of great significance to explore the methods to mitigate the toxicity of heavy metals in livestock manure. As a simple and economic solution, composting has been proved to be effective in immobilization of heavy metals, which can reduce heavy metals bioavailability by changing their speciation and mitigating their mobility^3^.

The biochemical reaction during process of composting has been confirmed to play a crucial role in the immobilization of heavy metals. Leita and De Nobili^4^ first reported the evolution of water-extractable heavy metals during composting and found that composting could effectively reduce bioavailability of Pb and Zn. After that, scientists attempted to reduce heavy metals bioavailability more significantly by regulating the composting process. Therefore, the effects of parameters that can directly regulate the composting process and compost quality on the changes of heavy metal speciation during composting have been widely investigated, such as aeration rate, moisture content, C/N ratio, and bulking agent type^5,6^. In practice, modulating C/N ratio and bulking agent type was widely acceptable as frequently-used measurement to reinforce mitigating heavy metals bioavailability.

With the help of mathematical analysis methods, present work aims to explore the key influencing factors on heavy metals bioavailability during the basic process of composting by regulating the initial C/N ratio and selecting bulking agent type. The initial C/N ratio is one of the most crucial factors influencing composting process and compost quality, which can affect the nitrogen loss and the thermophilic phase duration during composting and thus affect the maturity and stability of compost^7,8^. Due to the high moisture content, low C/N ratio, and high density of livestock manure, bulking agents are usually used in composting to adjust the physical properties of livestock manure^9^. Different bulking agents can provide different sources of organic matter, also adjust the physicochemical properties of compost, such as porosity, pH, electrical conductivity, and cation exchange capacity, so as to regulate the nitrogen loss, organic matter degradation and stabilization during composting^10,11^. Both initial C/N ratio and bulking agent can significantly influence the composting process, and more importantly, they are easy to be adjusted and changed. As a multivariate analysis method, RDA is more widely used to relate microbial community changes with environmental changes^12^, but it also has great application potential in analyzing the correlation between heavy metal speciation and composting related physicochemical parameters.

Numerous researches on the factors influencing heavy metal bioavailability during composting have shown that the bioavailability of heavy metals is mainly affected by organic matter, pH^13,14^ and various other factors. However, most of them only considered a limited range of composting related physicochemical parameters^13,15^, which were not comprehensive enough to characterize the processing properties of composting. In addition, previous studies on the mechanism of heavy metal immobilization during composting rarely considered the effect of initial C/N ratio and bulking agent type, although Wang et al.^3^ explored the immobilization mechanism of Cu and Zn during composting under various initial C/N ratios.

Therefore, the specific aims of this work were to evaluate the effects of different initial C/N ratios and bulking agent types on multiple physicochemical parameters related to composting and the bioavailability of Cd, As and Cr. Meanwhile, mathematical analysis methods including redundancy analysis (RDA) were employed to identify the key factors influencing the redistribution of Cd, As and Cr fractions during swine manure composting with different initial C/N ratios and bulking agent types, and the immobilization mechanisms of different heavy metals were also discussed. It was hypothesized that (1) different initial C/N ratios and bulking agent types would affect the relevant physicochemical parameters through regulating the composting process, and the changes of physicochemical parameters would further influence the bioavailability of Cd, As and Cr; (2) the immobilization mechanism of different heavy metals could be different, and humic substances complexation, phosphate precipitation, and biosorption may play a critical role in the reaction process. These results will provide theoretical guidance for the immobilization of Cd, As and Cr by composting technology.

## Results and discussion

### Changes in physicochemical parameters

**Figure 1:**
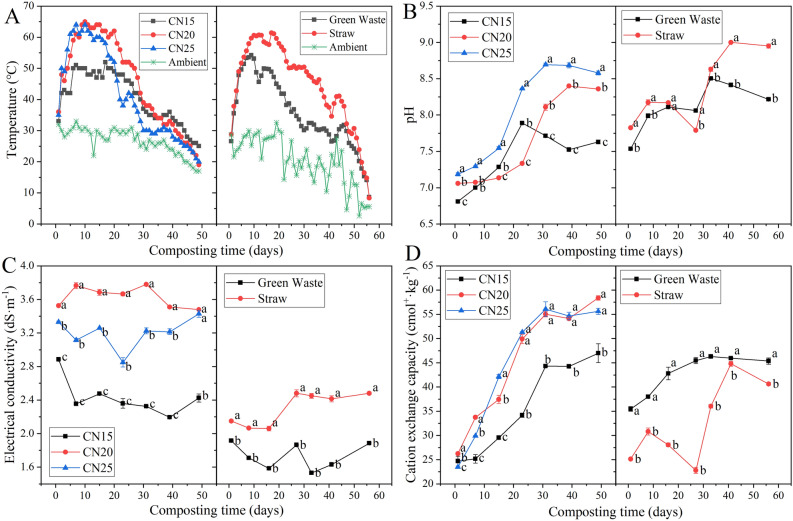
Changes in temperature (**A**), pH (**B**), electrical conductivity (**C**), and cation exchange capacity (**D**) during swine manure composting with different initial C/N ratios and bulking agent types. Different lowercase letters indicate significant differences among treatments, while the same lowercase letters indicate no significant differences among treatments (*p* < 0.05). The error bars represent the standard deviation of three replicates.

The evolution of temperature in different treatments is shown in Fig. 1A. For the five treatments C/N_15_, C/N_20_, C/N_25_, GW and ST, the highest temperature reached 52 °C, 65 °C, 64 °C, 54 °C and 61 °C, and the thermophilic phase (> 50 °C) lasted for 5 d, 24 d, 19 d, 5 d and 26 d, respectively. The temperature profile showed that higher C/N ratio (20:1 or 25:1) and straw as bulking agent were helpful for composting to achieve higher temperature and longer period of thermophilic phase. During composting, the pH of all treatments showed a fluctuating rising trend (Fig. 1B). The increase in pH during composting was generally caused by the degradation of organic acids and the absorption of ammonia produced by the decomposition of nitrogen-containing organic compounds. However, when organic compounds decompose to produce organic acids, it will lead to the decrease of pH^13,16^. The increase of pH in all treatments was as follows: C/N_25_ > C/N_20_ > C/N_15_, ST > GW. Results showed that compared with green waste, straw could better promote the increase of pH value. Meanwhile, the treatment with higher initial C/N ratio was more conducive to the adsorption and accumulation of ammonia due to its higher straw content, leading to a greater increase in pH value. EC reflects the concentration of soluble salts in compost. It can be seen from Fig. 1C that the EC of ST was significantly higher than that of GW, which may be due to the higher content of soluble salts in straw in comparison with green waste. After composting, the EC of C/N_15_ and ST decreased and increased slightly, respectively, while the EC of the other treatments showed no significant change. CEC reflects the ability of compost to absorb heavy metal cations^17^, which is usually positively correlated with humification degree^18^. The CEC of all treatments showed an increasing trend during composting (Fig. 1D), among which the CEC of C/N_20_, C/N_25_ and ST increased the most. The reason may be that high initial C/N ratio and straw as bulking agent are more conducive to the humification process during composting.

**Figure 2:**
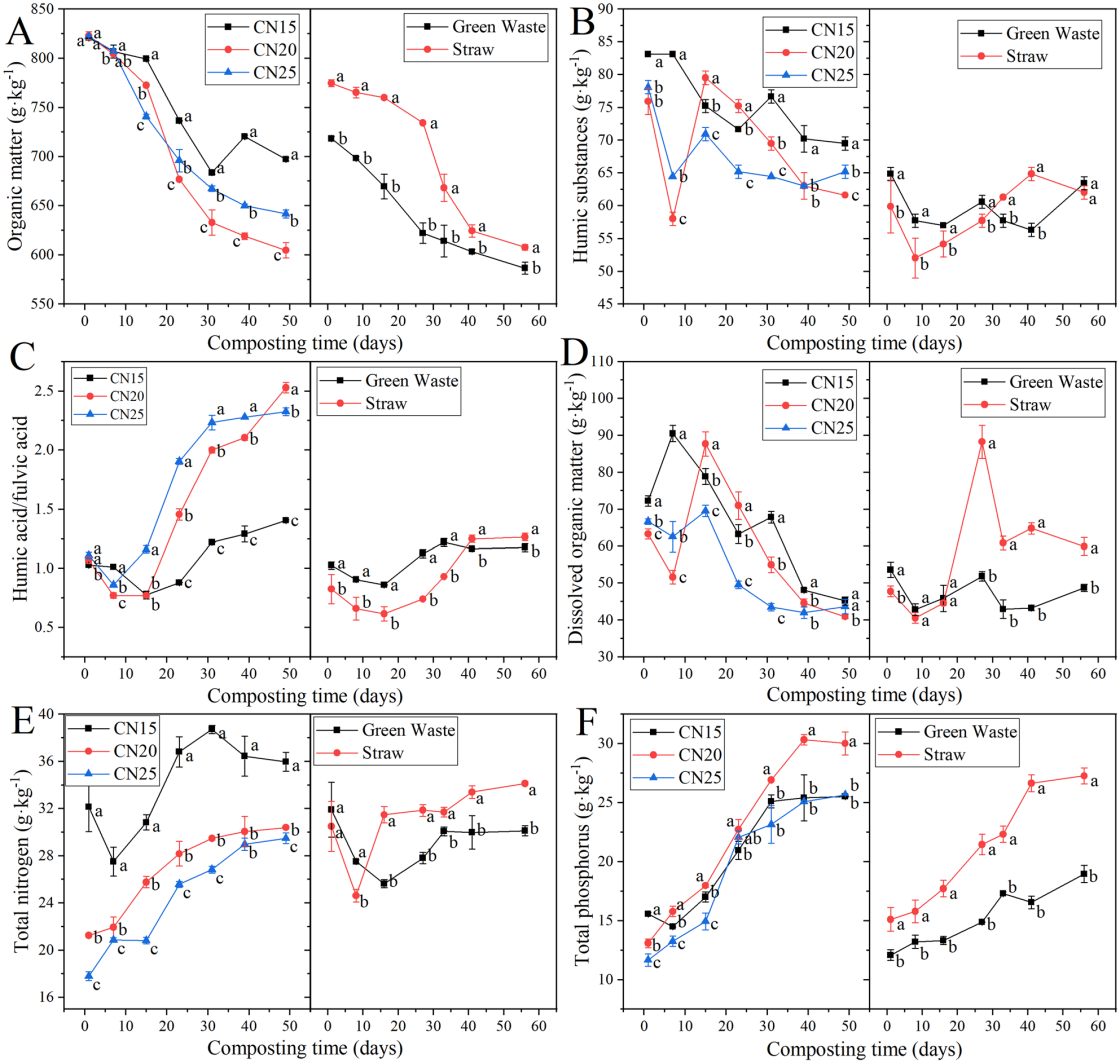
Changes in organic matter (**A**), humic substances (**B**), humic acid/fulvic acid (**C**), dissolved organic matter (**D**), total nitrogen (**E**), and total phosphorus (**F**) during swine manure composting with different initial C/N ratios and bulking agent types. Different lowercase letters indicate significant differences among treatments, while the same lowercase letters indicate no significant differences among treatments (*p* < 0.05). The error bars represent the standard deviation of three replicates.

The changes of OM in different treatments followed the same trend (Fig. 2A), which decreased rapidly in the first 30 days, and then gradually stabilized. The total OM degradation after composting followed the order: C/N_20_ > C/N_25_ > C/N_15_, ST > GW, indicating that higher initial C/N ratio and straw as bulking agent could better facilitate the degradation of OM. DOM is an important component of organic matter, which is composed of HS and non-HS, and HS comprises two main fractions, HA and FA. The humification degree of compost can be effectively characterized by HA/FA^15^. It can be seen from Fig. 2B that the HS of C/N_15_, C/N_20_ and C/N_25_ decreased significantly after composting, while that of GW and ST showed no significant change. The changes of HA/FA were found similarly among different treatments (Fig. 2C), showing a trend of decreasing first, then increasing, and then gradually stabilizing. After composting, the HA/FA in all treatments increased as follows: C/N_20_ > C/N_25_ > C/N_15_, ST > GW, indicating that C/N_20_ and ST had the highest degree of humification. Except for ST, the DOM in other treatments all showed a certain degree of decline after composting (Fig. 2D). Regarding the change of TN (Fig. 2E) and TP (Fig. 2F) during composting, the TN of C/N_15_, ST and GW first decreased and then increased, while the TN and TP of the other treatments showed a steady increasing trend. After composting, the TN and TP of C/N_20_ and ST showed the most significant increase, indicating that an initial C/N ratio of 20:1 and straw as bulking agent was more beneficial to the improvement of nitrogen and phosphorus content, resulting in higher nutritional value and economic benefits of compost products.

### Redistribution of Cd, As and Cr fractions during composting

**Figure 3:**
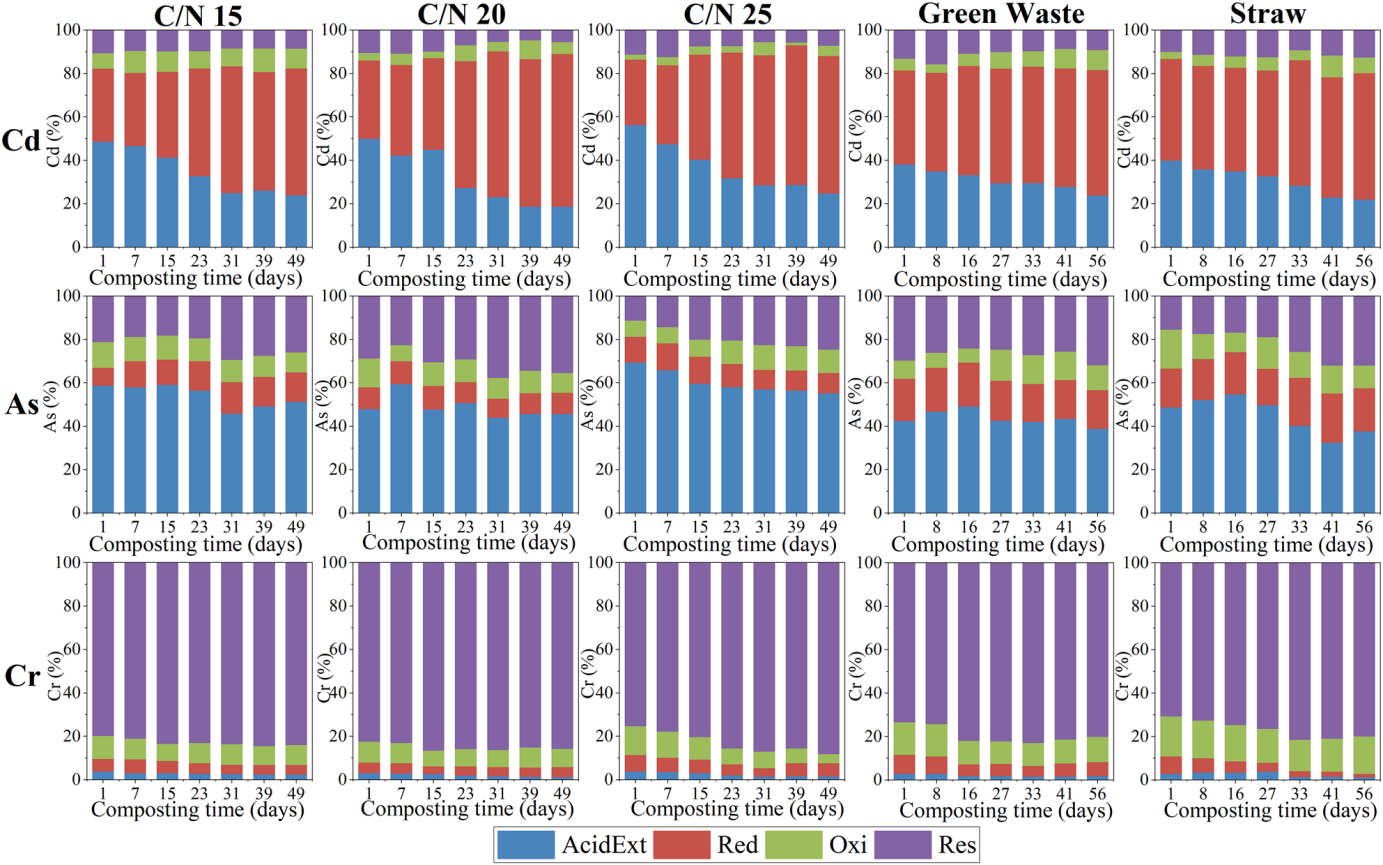
Redistribution of Cd, As and Cr fractions during swine manure composting with different initial C/N ratios and bulking agent types. *AcidExt* acid extractable fraction, *Red* reducible fraction, *Oxi* oxidizable fraction, *Res* residual fraction.

The redistribution of Cd, As and Cr fractions during pig manure composting is shown in Fig. 3. The AcidExt-Cd and Red-Cd accounted for over 80% of the total Cd in the composts, implying a great potential environmental threat of Cd if changes occur in the external conditions such as pH and redox potential^19^. As composting proceeded, Cd was mainly transformed from the AcidExt fraction to the Red fraction. After composting, the BF-Cd did not change significantly, while the proportion of the AcidExt-Cd in C/N_15_, C/N_20_, C/N_25_, GW and ST decreased by 50.7%, 62.4%, 56.0%, 37.7% and 44.9%, respectively. Compared with Cd, the proportion of Res-As was relatively higher, indicating that the mobility of As was weaker than that of Cd. During composting, the proportion of AcidExt-As gradually decreased, while the proportion of Res-As continued to increase. The reduction of BF-As in C/N_15_, C/N_20_, C/N_25_, GW and ST treatments was 3.1%, 4.2%, 20.6%, 8.5% and 13.6%, respectively. For Cr, the Oxi and the Res fractions were dominant speciation in all treatments, which implied a low potential environmental risk. After composting, the BF-Cr in C/N_15_, C/N_20_, C/N_25_, GW and ST decreased by 29.9%, 26.2%, 32.2%, 27.6% and 73.6%, respectively.

The results indicated that an initial C/N ratio of 25:1 was beneficial for reducing the bioavailability of As and Cr. For Cd, although there was no significant change in BF after composting, the AcidExt fraction with the strongest mobility was greatly reduced, indicating that the composting process still had certain immobilization effect on Cd, and an initial C/N ratio of 20:1 was more favorable for its immobilization. Similar results were reported by Wu et al.^5^ that an initial C/N ratio of 25 better reduced the bioavailability of Cu and Zn among treatments with C/N ranging from 15 to 25. The reason can be attributed to that relatively higher C/N ratio is beneficial to improve the OM conversion process and accelerate the OM degradation and humification, thus strengthening the immobilization effect of heavy metals^5,16^. In addition, result shows that using straw as bulking agent was more effective in reducing the bioavailability of Cd, As and Cr. The reason may be that, compared with green waste, the loose structure of straw is more conducive to aeration, which could lead to a better composting effect^6^.

### Redundancy analysis of physicochemical parameters and speciation of Cd, As and Cr

**Figure 4:**
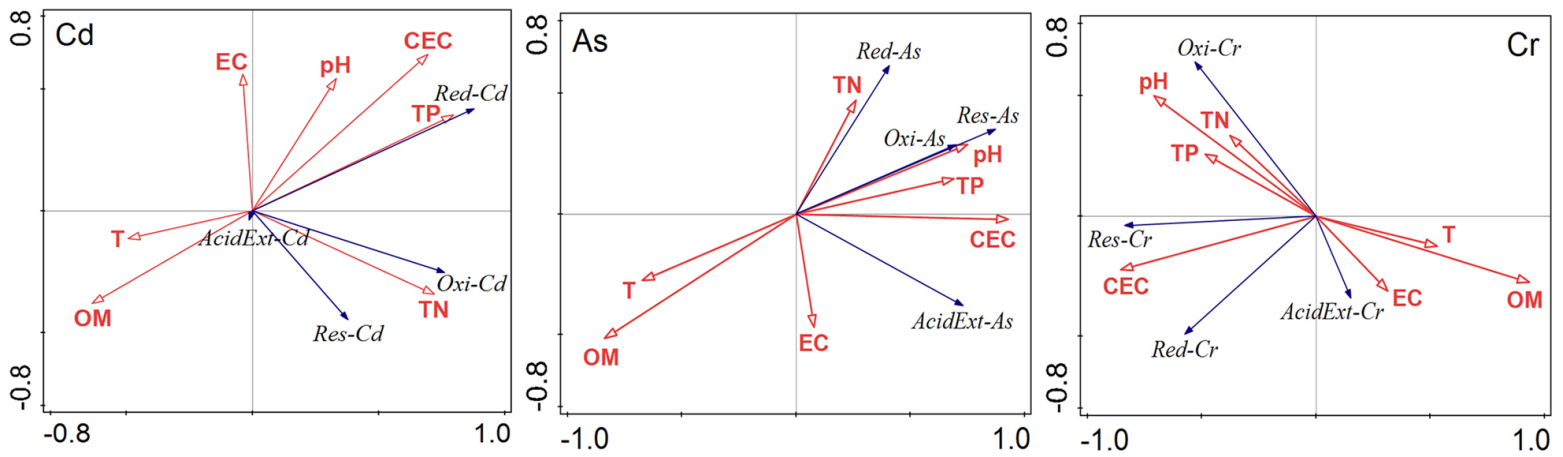
Redundancy analysis of physicochemical parameters and speciation of Cd, As and Cr during swine manure composting with different initial C/N ratios and bulking agent types. *OM* organic matter, *EC* electrical conductivity, *CEC* cation exchange capacity, *T* temperature, *TP* total phosphorus, *TN* total nitrogen, *AcidExt* acid extractable fraction, *Red* reducible fraction, *Oxi* oxidizable fraction, *Res* residual fraction.

In order to reveal the interaction between the physicochemical properties of compost and the bioavailability of heavy metals, seven physicochemical parameters (temperature, pH, EC, CEC, OM, TN, TP) were selected for RDA. The results of RDA are shown in Fig. 4 and Table 1. It can be seen from Fig. 4 that OM was significantly negatively correlated with TP, TN, and heavy metals, which could be attributed to the increase of non-volatile element residue caused by OM loss during composting^3^.

**Table 1:** Simple explanation quantity (SEQ) and conditional explanation quantity (CEQ) of physicochemical parameters on the redistribution of Cd, As and Cr fractions during swine manure composting with different initial C/N ratios and bulking agent types.

Cd			As			Cr		
Parameters	SEQ (%)	CEQ (%)	Parameters	SEQ (%)	CEQ (%)	Parameters	SEQ (%)	CEQ (%)
TP	37.4**	37.4**	CEC	49.8**	49.8**	OM	36.4**	36.4**
TN	30.9**	11.1**	OM	44.5**	14.8**	CEC	31.7**	15.5**
pH	12.1**	9.0**	pH	34.6**	5.7**	EC	12.2**	10.3**
CEC	31.4**	6.9*	T	27.2**	4.7**	TP	15.9**	5.8**
EC	5.0*	5.7**	EC	6.7**	2.2	pH	25.4**	2.5
OM	26.3*	2.1	TN	7.5	1.1	T	12.0**	1.5
T	13.9	0.7	TP	28.5	0.2	TN	9.1*	0.3

*Statistically significant at the probability level 0.05.

**Statistically significant at the probability level 0.01.

Table 1 shows the quantitative results of RDA. From the SEQ, it can be known that the physicochemical parameters with the highest explanation quantity for the redistribution of Cd, As and Cr fractions to be TP (37.4%), CEC (49.8%), and OM (36.4%), respectively. Meanwhile, the CEQ, taking into account combinations of factors, found that TP and TN, CEC and OM, and OM, CEC and EC explained 48.5%, 64.6% and 62.2% of the redistribution of Cd, As and Cr fractions, respectively, which is significantly higher than using only one factor to explain. Results demonstrate that the bioavailability of Cd, As and Cr were significantly correlated with total N and P, CEC and OM, and OM, CEC, and EC, respectively.

CEC represents the negative charge that exists on the surfaces of clay and OM, which can effectively reflect the ability of compost to adsorb heavy metal cations^17^. In many cases, heavy metal sorption occurs through non-specific sorption processes. CEC explains the affinity adsorption of heavy metals by compounds present in the compost other than OM, especially iron oxide and carbonate^20^. Therefore, to some extent, CEC explains the reduction in bioavailability of heavy metals during composting. In this study, over 50% of the redistribution information of As and Cr fractions could be well explained by the combination of OM and CEC. The results indicate that the speciation of As and Cr was likely to be influenced by both organic and inorganic substances.

The results of RDA showed that TP and TN were important factors influencing the speciation of Cd. With the progress of composting, the increase of TP concentration may improve the possibility of Cd to form cadmium phosphate precipitation, or co-precipitate with other phosphates^13,21^, thus reducing the mobility and bioavailability of Cd. Another explanation of this result could be the biosorption function played by the microorganisms^22^. Biosorption can adsorb heavy metal ions on the surface of microorganisms as well as provide nucleation sites for the formation of stable heavy metal minerals, thus immobilize heavy metals^23^. Studies have shown that microbial biomass, species and diversity can significantly affect the biosorption of heavy metal ions^22,24^. Furthermore, P and N can regulate microbial metabolism and thus affecting microbial community and biomass^3,25^. During composting, the contents of TN and TP had an obvious increase, which might affect the biomass and diversity of microorganisms through regulating microbial metabolism, and further affect the biosorption efficiency of Cd. In the present study, TN and TP together could explain 48.5% of the redistribution information of Cd fractions, while OM could only explain 26.3% of it, indicating that the immobilization of Cd during composting could mainly be attributed to the formation of phosphate precipitation and biosorption.

EC represents the total inorganic salt ions of compost^26^, which is one of the important indicators to influence process reaction during composting except for organic matter. Previous studies have shown that an increase in salinity could elevate heavy metals bioavailability. Liu et al.^27^ found that higher levels of salinity could increase the bioavailable fractions of Cd and Cr to a greater extent than lower levels of salinity. Zhao et al.^28^ reported that an increase in salinity promoted the desorption and release of Cd by enhancing the chloro-complexation and elevating the acidity, respectively, thus promoting the bioavailability of Cd. The results of RDA show that, in addition to OM and CEC, EC also had a significant correlation with the redistribution of Cr fractions, demonstrating that the mechanism of Cr speciation changes could be relatively complex. The bioavailability of Cr was influenced by the combined effects of organic–inorganic factors such as OM, iron/aluminum oxide, inorganic salt.

### Correlation between OM components and bioavailability of Cd, As and Cr

**Figure 5:**
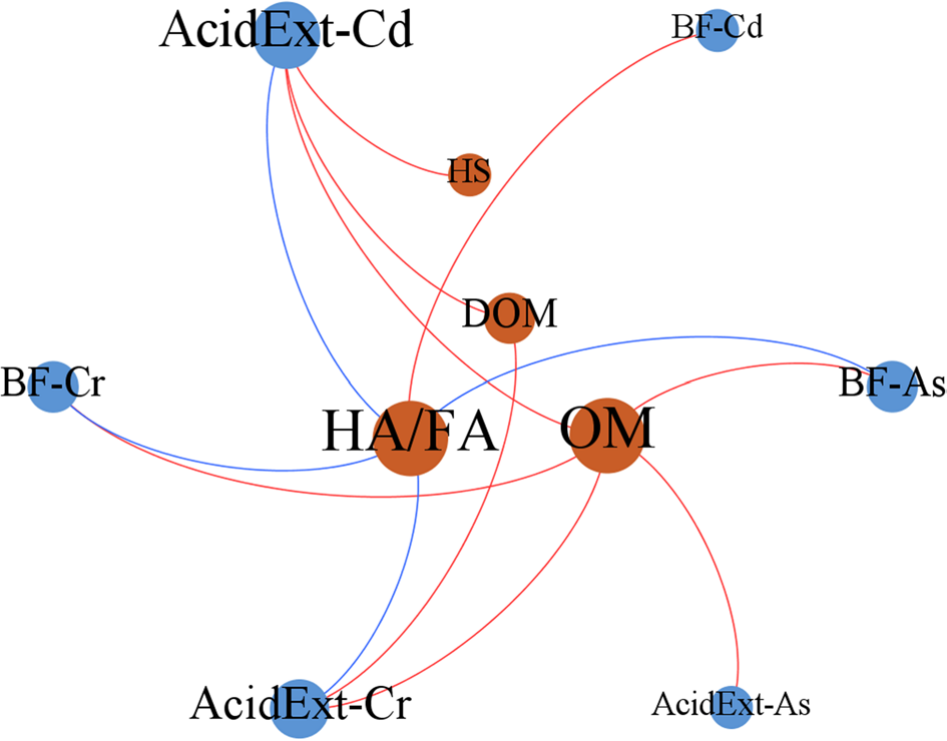
Network analysis shows the relationship between different components of organic matter and bioavailability of Cd, As and Cr during swine manure composting with different initial C/N ratios and bulking agent types. Connections between nodes represent significant correlation (*p* < 0.05) by Pearson's correlation model. The red and blue connections indicate positive and negative correlation, respectively. Elements with a larger node sizes have a greater relationship with the other components. *OM* organic matter, *HS* humic substances, *HA/FA* humic acid:fulvic acid ratio, *DOM* dissolved organic matter, *AcidExt* acid extractable fraction, *BF* bioavailability factor.

To further explore the mechanism of the key influencing factor, OM, on the redistribution of Cd, As and Cr fractions, the correlation between OM, HS, HA/FA, DOM, and the AcidExt fraction and BF (sum of the AcidExt and Red fractions) of Cd, As and Cr were analyzed (Fig. 5). It could be seen from Fig. 5 that OM was positively correlated with AcidExt-Cd, As, Cr and BF-As, Cr; DOM was positively correlated with AcidExt-Cd, Cr; HA/FA was negatively correlated with AcidExt-Cd, Cr and BF-As, Cr. However, the correlation between HS and Cd, As, Cr was relatively weak.

The results indicated that DOM has a significant promotion effect on the AcidExt fraction of heavy metals, while the humification process which characterized by HA/FA has a significant inhibitory effect on the bioavailability of heavy metals. Studies have reported that DOM, especially low molecular weight organic acids, can increase the mobility and bioavailability of heavy metals by forming soluble chelates with heavy metals^29,30^. However, HS has a large number of functional groups such as carboxyl and hydroxyl groups, which can easily form stable complexes with heavy metals to reduce its mobility^3^. In addition, HA has higher molecular weight, more condensed structure and relatively lower water solubility than FA^15^, which results in more stable complexation of heavy metals with HA than with FA. In the present study, it is clear that the higher values of AcidExt-Cd and Cr found in the early stages of composting were related to the abundance of soluble forms of OM, which is shown by the positive correlation with DOM. With the gradual degradation and stabilization of OM during composting, the relative content of AcidExt-Cd and Cr decreased as a consequence of higher retention capacity of the more humified OM, which is shown by the positive correlation with total OM but negative with HA/FA. Moreover, incremental HA/FA and pH during composting were conducive to the complexation of heavy metals with HA^3^, thus promoting the transformation of heavy metals into more stable forms.

## Conclusions

Compared with low initial C/N ratio (15:1) and green waste as bulking agent, higher initial C/N ratio (20:1 and 25:1) and straw were more conductive to decreasing bioavailability of Cd, As and Cr during swine manure composting. The bioavailability of Cd was significantly correlated with TN and TP, while that of As and Cr were more susceptible to OM, CEC and OM, CEC, EC, respectively, implying that the immobilization of Cd could mainly be attributed to the formation of phosphate precipitation and biosorption, while the speciation of As and Cr was mainly influenced by the joint impact of organic–inorganic factors. Further analysis of organic components showed that DOM and humification degree were significantly associated with the changes of Cd, As and Cr bioavailability. In summary, relative higher initial C/N ratio and bulking agent of straw contributed to the decrease of Cd, As and Cr bioavailability during composting, in which phosphate precipitation, biosorption and humification processes might be involved.

## Materials and methods

### Experimental procedure and sampling

**Figure 6:**
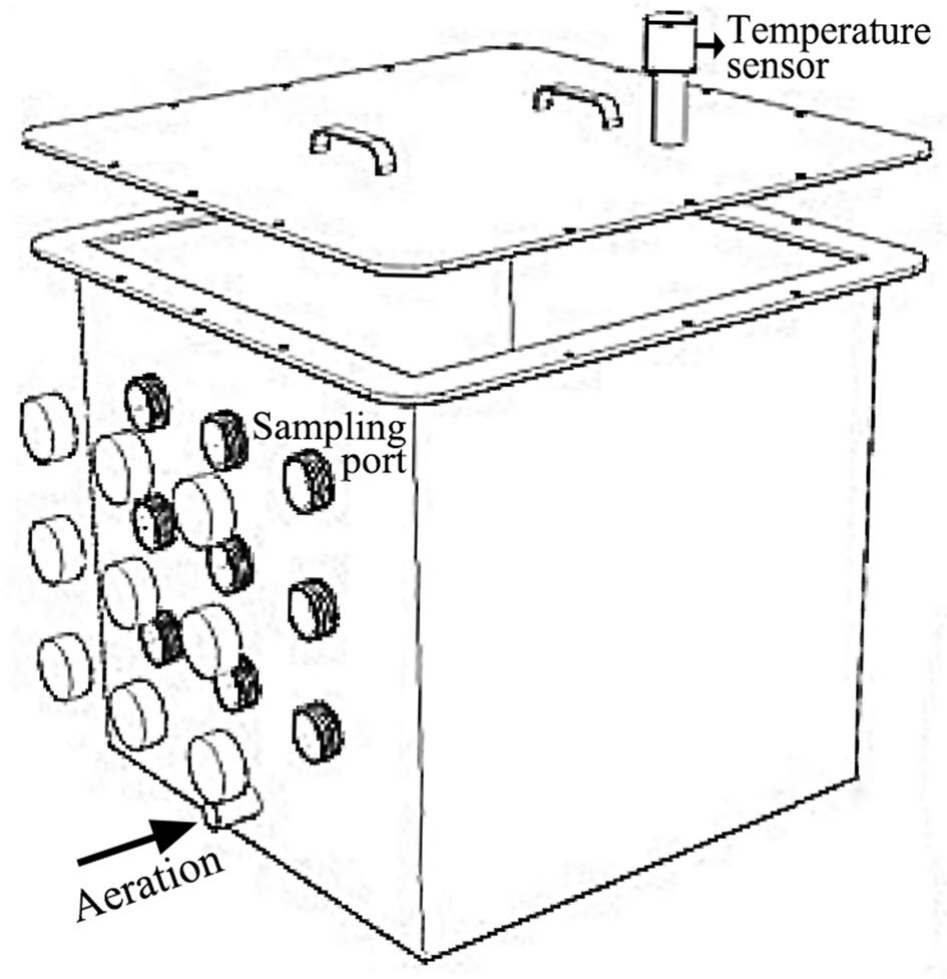
Schematic diagram of the composting reactor.

Fresh swine manure was used as the main compost raw material. For treatments with different initial C/N ratios, rice straw (smashed to a diameter of 1–2 cm) was used as the bulking agent to adjust the initial C/N ratio to 15:1 (C/N_15_), 20:1 (C/N_20_), and 25:1 (C/N_25_). For treatments with different bulking agent types, green waste (GW) and rice straw (ST) (smashed to a diameter of 1–2 cm) were used as bulking agents to adjust the initial C/N ratio to 15:1. The physicochemical properties of composting raw materials are shown in Table 2. The mixtures (about 150 kg each) were composted in self-made polyvinyl chloride reactors with a volume of 640 L (0.8 m × 0.8 m × 1.0 m, length × width × height), equipped with an automatic aeration device and an automatic temperature sensor (Fig. 6). The initial water content was adjusted to 65%. Continuous aeration was supplied at a frequency of 20 min/h, and the aeration volume was controlled at 6 m^3^/h. During sampling, the sub samples at 9 different positions of the reactor were collected and mixed to form the representative sample. Composite samples of treatments with different initial C/N ratios and bulking agent types were collected on days 1, 7, 15, 23, 31, 39, 49 and 1, 8, 16, 27, 33, 41, 56, respectively, then air dried, ground and sieved for further analysis.

**Table 2:** Physicochemical properties of composting raw materials (dry weight).

Parameters	Swine manure	Rice straw	Green waste
OM (g/kg)	782.22 ± 7.38	874.50 ± 10.94	667.01 ± 21.35
TN (g/kg)	33.60 ± 0.64	10.74 ± 0.12	7.84 ± 0.96
TP (g/kg)	15.8 ± 0.23	3.63 ± 0.10	2.94 ± 0.18

### Analytical methods

Three replicates were used in all parameters analysis. Temperature was automatically monitored and recorded by the automatic temperature sensor installed in the composting reactor. The pH and electrical conductivity (EC) were measured in water extracts at a compost:water ratio of 1:10 (w/v). Organic matter (OM) was determined by measuring the combustion loss at 550 °C for 8 h^13^. Cation exchange capacity (CEC) was determined by the hexamminecobalt trichloride solution-spectrophotometric method (China, HJ 889-2017)^31^. Total nitrogen (TN) was determined by the modified Kjeldahl method (China, HJ 717-2014)^32^. Total phosphorus (TP) was determined by the alkali fusion-Mo-Sb anti-spectrophotometric method (China, HJ 632-2011)^33^. Humic substances (HS) was extracted by shaking with 0.1 M NaOH at 25 °C for 24 h. The above extract was acidified to pH 1 with 3 M H_2_SO_4_, allowed to stand overnight, and fulvic acid (FA) was assessed from the supernatant after centrifuging. Humic acid (HA) was calculated by subtracting the FA from the HS^34^. Dissolved organic matter (DOM) was extracted with 0.5 M K_2_SO_4_, and the resulting solution was filtered through a 0.45 μm filter and analyzed by total organic carbon analyzer (Apollo 9000, Tekmar–Dohrmann, USA).

The speciation of Cd, As and Cr were determined by the modified Community Bureau of Reference sequential extraction method reported by Nemati et al.^35^, in which metals were partitioned into acid extractable (AcidExt), reducible (Red), oxidizable (Oxi) and residual (Res) fractions. The AcidExt fraction was extracted with 0.11 M CH_3_COOH by shaking the compost sample at 25 °C for 16 h. The Red fraction was extracted with 0.5 M NH_2_OH·HCl (adjusted to pH 1.5 with HNO_3_) by shaking the residue from the first step at 25 °C for 16 h. To extract the Oxi fraction, 8.8 M H_2_O_2_ was used to oxidize the residue from the previous step twice, then 1.0 M CH_3_COONH_4_ was added to the residue and shaken at 25 °C for 16 h. The Res fraction was obtained by digesting the residue from the previous step with HNO_3_ and HClO_4_. The extract was filtered through a 0.45 μm filter. The contents of Cd and Cr in the extract were analyzed by inductively coupled plasma optical emission spectrometry (Optima 5300DV, Perkin-Elmer, USA) and As was analyzed by atomic fluorescence spectrometer (PSA 10.825, P S Analytical, UK). The bioavailability factor (BF) of heavy metals was defined as the sum of the ratios of the AcidExt and Red fractions contents to the total content^36^.

### Statistical analysis

The general statistical analysis was completed using SPSS 25.0, and redundancy analysis (RDA) was performed using the Canoco software (version 5.0, released in 2012, USA). The simple explanation quantity (SEQ) in the RDA results is the explanation quantity measured when physicochemical parameters are separately imported, while the conditional explanation quantity (CEQ) is the increased explanation quantity measured when physicochemical parameters are continuously added according to the contribution degree. General figures were drawn using OrginPro (version 2018C, OriginLab Corporation, Northampton, MA, USA). The network analysis diagram was performed using Gephi (version 0.9.2, released in 2017, USA).
